# *AP5Z1/SPG4*8 frequency in autosomal recessive and sporadic spastic paraplegia

**DOI:** 10.1002/mgg3.87

**Published:** 2014-05-25

**Authors:** Nina A Schlipf, Rebecca Schüle, Sven Klimpe, Kathrin N Karle, Matthis Synofzik, Julia Wolf, Olaf Riess, Ludger Schöls, Peter Bauer

**Affiliations:** 1 Institute of Medical Genetics and Applied Genomics, University of TübingenTübingen, Germany; 2 Institute of Human Genetics, University Medical Center FreiburgFreiburg, Germany; 3 Clinical Neurogenetics, Department of Neurology and Hertie-Institute for Clinical Brain Research, University of TübingenTübingen, Germany; 4 Department of Neurology, University of MainzMainz, Germany; 5 German Center for Neurodegenerative Diseases (DZNE)Tübingen, Germany

**Keywords:** *AP5Z1*, hereditary spastic paraplegia, SPG48, targeted next-generation sequencing

## Abstract

Hereditary spastic paraplegias (HSP) constitute a rare and highly heterogeneous group of neurodegenerative disorders, defined clinically by progressive lower limb spasticity and pyramidal weakness. Autosomal recessive HSP as well as sporadic cases present a significant diagnostic challenge. Mutations in *AP5Z1*, a gene playing a role in intracellular membrane trafficking, have been recently reported to be associated with spastic paraplegia type 48 (SPG48). Our objective was to determine the relative frequency and clinical relevance of *AP5Z1* mutations in a large cohort of 127 HSP patients. We applied a targeted next-generation sequencing approach to analyze all coding exons of the *AP5Z1* gene. With the output of high-quality reads and a mean coverage of 51-fold, we demonstrated a robust detection of variants. One 43-year-old female with sporadic complicated paraplegia showed two heterozygous nonsynonymous variants of unknown significance (VUS3; p.[R292W];[(T756I)]). Thus, *AP5Z1* gene mutations are rare, at least in Europeans. Due to its low frequency, systematic genetic testing for *AP5Z1* mutations is not recommended until larger studies are performed to add further evidence. Our findings demonstrate that amplicon-based deep sequencing is technically feasible and allows a compact molecular characterization of multiple HSP patients with high accuracy.

## Introduction

Autosomal recessive hereditary spastic paraplegia (ARHSP) is a clinically and genetically heterogeneous neurodegenerative disorder characterized by progressive lower limb spasticity and pyramidal weakness because of axonal degeneration of the corticospinal tracts and dorsal columns. According to the presence of additional neurological signs, including cerebellar ataxia, peripheral neuropathy, epilepsy, optic neuropathy, and intellectual disability, ARHSPs are distinguished into pure and complex forms. Genetically, several ARHSP loci and at least 40 disease-associated genes have been identified (Fink 2013; Novarino et al. 2014). It is well known that the most frequent causes of ARHSP are mutations in the gene *SPG11* (Online Mendelian Inheritance in Man [OMIM] no. 610844) (Stevanin et al. 2007). ARHSP families negative for such mutations present a significant diagnostic challenge. For a cost- and time-efficient diagnostic routine, information about the frequency of newly identified hereditary spastic paraplegia (HSP) genes is necessary. Techniques like next-generation sequencing (NGS), with its massively parallel and high throughput, increase the potential to analyze clinically relevant genes, in order to identify mutations.

Slabicki et al. (2010) reported in two French siblings a homozygous indel mutation in exon 2 (p.R27Lfs*3) of the *AP5Z1* gene (OMIM 613653), encoding adaptor protein complex 5 zeta 1(AP1Z1), as the underlying genetic cause of autosomal recessive SPG48 (OMIM 613647). Both siblings have pure adult-onset spastic paraplegia with hyperintensity of the cervical spinal cord in one sibling as the only distinguishing magnetic resonance imaging (MRI) feature (Slabicki et al. 2010). Recently, Novarino et al. (2014) identified another homozygous *AP5Z1* mutation (p.L701P) in a single family displaying pure ARHSP. *AP5Z1* forms a subunit of the adaptor protein complex 5 (AP-5), which is associated with the known ARHSP-associated proteins spatacsin (SPG11) and spastizin (SPG15). *AP5Z1* is involved in membrane trafficking and appears to be the best candidate for endosomal sorting (Hirst et al. 2011). The frequency of SPG48 among apparently sporadic or ARHSP cases as well as its associated phenotype is unknown, as no further families with *AP5Z1* mutations have been described so far. To study the frequency and the phenotype of SPG48, we performed a molecular screening investigating *AP5Z1* in a cohort of 127 HSP patients of Caucasian origin. Furthermore, we demonstrated an amplicon-based NGS strategy that is feasible and allows a molecular characterization of multiple HSP patients in a massive way with high accuracy.

## Materials and Methods

We set out to investigate the frequency of *AP5Z1* (RefSeq accession number: NM_014855.2) mutations as a cause of ARHSP. To this, a consecutive series of 127 index patients (39 pure form, 88 complex form), including 96 sporadic and 31 HSP cases compatible with autosomal recessive inheritance, were recruited through the German Network for Hereditary Movement Disorders and the Tübingen HSP outpatient clinic. All patients were of European descent. In all patients with either cognitive deficits (*n* = 9) or *corpus callosum* dysgenesis on MRI (*n* = 6) or both, mutations in the *SPG11* (OMIM 610844) gene as well as the *ZFYVE26* gene (OMIM 612012) were excluded. Mutations in the *CYP7B1* (OMIM 603711) and *SPG7* gene (OMIM 602783) have been excluded in all cases. We used an amplicon-based NGS strategy for barcoding and multiplexing thousands of PCR amplicons for deep sequencing onto the Roche 454 NGS platform (454 Life Sciences, Branford, CT). All patients were screened for gene dosage in the *AP5Z1* gene using a multiplex ligation-dependent probe amplification assay. For amplicon-library generation conditions, primer sequences (Tables S1 and S2), copy number variation analysis, and data analysis procedure including variation interpretation see supporting information.

## Results and Discussion

An array-based amplification strategy followed by NGS was used to detect *AP5Z1* mutations in a cohort of 127 patients representing sporadic or recessive HSP. We performed the amplification of target regions on a microfluidic system (Fluidigm 48.48 AccessArray™ System, Fluidigm Corporation, San Francisco, CA) and processed the emulsion-based clonal amplification and sequencing protocol using the medium-volume GS FLX Titanium amplicon workflow (454 Life Sciences). Overall, a median of 74,289 high-quality sequencing reads (passed filter wells) were generated per patient pool (48 PCR amplicons and 48 study samples). The median coverage per amplicon was 51-fold, ranging from 12- to 164-fold (mean coverage 107-fold). Both, the forward and reverse strands, were successfully and homogeneously sequenced as demonstrated in Figure S1. The median length of reads per patient pool was 334 bp. Per patient, the median of base pairs sequenced ranged from 413 to 1040 kbp. Dropouts of single amplicons with no coverage were observed in 91 (4.2%) of 2159 PCR products. Furthermore, 7.8% of the amplicons (169 of 2159) were insufficiently covered with less than 10 reads (Figure S2). All amplicons without any coverage or covered less than 10-fold were additionally analyzed by conventional Sanger sequencing.

Co-occurrence of two mutations as expected in the recessive *AP5Z1* gene was identified in only one patient. The 43-year-old female with sporadic complicated paraplegia showed two heterozygous nonsynonymous variants of unknown significance (VUS3; c.874C>T [p.R292W] and c.2267C>T [p.T756I]). Interpretation of these variants is summarized in Table 1. Unfortunately, no further family members were available in order to establish the chromosomal status of both variants. The patient presented a sporadic complicated HSP and showed cerebellar affection manifesting as myokymia and congenital bilateral nystagmus. Brain MRI was normal. The reported SPG48 phenotype represents a complicated adult-onset SPG with urinary incontinence, normal brain MRI, and hyperintensities in the spinal cord in one patient (Slabicki et al. 2010). As only two families have been described so far, it is difficult to draw any genotype/phenotype correlations. Additionally, 17 known single-nucleotide polymorphisms (http://www.ncbi.nlm.nih.gov/SNP), 2 variants which had already been reported by Slabicki et al. (2010), 8 synonymous and 1 nonsynonymous single-nucleotide (p.S164G, heterozygous) variants, which were not considered as causative, were detected in our cohort (Table S3). We could not identify disease-causing mutations by gene dosage analysis.

**Table 1:** Nonsynonymous variants of unknown significance in the *AP5Z1* gene of patient with sporadic spastic paraplegia

Mutation at DNA level/protein level	Location	Heterozygous patients/homozygous patients	scorePhastCons^1^	scoreGERP^1^	Frequency sample chromosomes	Frequency “in-house” control chromosomes	dbSNP	Predicted effect (PolyPhen2)	Exome variant server (allele frequency)
c.874C>T/p.R292W	Exon07	1/0	0.051	2.87	1/266 (0.4%)	0/176 (0%)	rs199760184	Possibly damaging (score 0.456)	1/11,989 = 0.00008
c.2267C>T/p.T756I	Exon17	1/0	0.992	1.410	1/266 (0.4%)	0/146 (0%)	Not listed	Possibly damaging (score 0.667)	Not listed

*AP5Z1* RefSeq: NM_014855.2. GERP: “Distribution and intensity of constraint in mammalian genomic sequence.” Gregory M. Cooper et al. *Genome Res*. 2005.

1 Conservation scores: scorePhastCons (UCSC Genome Browser), scoreGERP (Genomic Evolutionary Rate Profiling, Sidow Lab).

These findings indicate a very low frequency of SPG48 in Europeans. With the output of high-quality reads and a mean coverage of 51-fold, we demonstrated a robust detection of variants. All sequence variants found in the patient cohort could be confirmed by Sanger sequencing. This indicates the high quality of our approach, furthermore, it demonstrates that our strategy is technically feasible and allows a compact molecular characterization of multiple HSP patients in a massive way with high accuracy. The diagnostic yield in our study cohort of ARHSP is still unclear; variants were identified but their pathogenicity is still elusive. Due to the low frequency of SPG48, we suggest that SPG48 should not be given a high priority when considering genetic screening for ARHSP mutations. Further studies are needed to fully understand the clinical relevance of *AP5Z1*, the frequency and relevance of mutations in Caucasian and non-Caucasian populations and to clarify the variants of unknown significance. Therefore, to address these issues we suggest in any case including *AP5Z1* in NGS gene panel diagnostics for ARHSPs.

## Supporting Information

**Figure S1.** Coverage distribution across amplicons. (A) For each of the amplicons (*x*-axis), the distribution of generated reads is represented (*y*-axis). Box-and-whiskers plots summarize the corresponding overall coverage and (B) according to forward (A reads) and reverse (B reads). (*AP5Z1* RefSeq: NM_014855.2). **Figure S2.** Performance of the study. In total, 88% of the amplicons (green, 1899 of 2159) were covered successfully (>10-fold). Dropouts of single amplicons with no coverage were obtained in 91 of 2159 amplicons (red, 4%) and 8% of the amplicons (blue, 169 of 2159) were insufficiently covered less than 10-fold and were completed by conventional Sanger sequencing (*AP5Z1* RefSeq: NM_014855.2). **Table S1.** Primers for all coding exons and intron boundaries of the *AP5Z1* gene (RefSeq: NM_014855.2). **Table S2.** Lib-A adaptor barcode primer. **Table S3.** Sequence variants found in *AP5Z1*.

## References

[b1] Fink JK (2013). Hereditary spastic paraplegia: clinic-pathologic features and emerging molecular mechanisms. Acta Neuropathol.

[b2] Hirst J, Barlow LD, Francisco GC, Sahlender DA, Seaman MN, Dacks JB (2011). The fifth adaptor protein complex. PLoS Biol.

[b3] Novarino G, Fenstermarker AG, Zaki MS, Hofree M, Silhavy JL, Heiberg AD (2014). Exome sequencing links corticospinal motor neuron disease to common neurodegenerative disorders. Science.

[b4] Slabicki M, Theis M, Krastev DB, Samsonov S, Mundwiller E, Junqueira M (2010). A genome-scale DNA repair RNAi screen identifies SPG48 as a novel gene associated with hereditary spastic paraplegia. PLoS Biol.

[b5] Stevanin G, Santorelli FM, Azzedine H, Coutinho P, Chomilier J, Denora PS (2007). Mutations in SPG11, encoding spatacsin, are a major cause of spastic paraplegia with thin corpus callosum. Nat. Genet.

