# Supplementary material for: AP5Z1/SPG48 frequency in autosomal recessive and sporadic spastic paraplegia
Source: Mol Genet Genomic Med. 2014 May 25;2(5):379–82. doi: 10.1002/mgg3.87 (PMC4190872; doi:10.1002/mgg3.87)
Supplement: Supplementary file 1 [file mgg30002-0379-SD1.doc]

***AP5Z1/SPG4*8 Frequency in Autosomal Recessive and Sporadic Spastic Paraplegia**

Nina A. Schlipf ^1,2,*^, Rebecca Schüle ^3,*^, Sven Klimpe ^4^, Kathrin N. Karle ^3, 5^, Matthis Synofzik ^3,5^, Julia Wolf ^3,5^, Olaf Riess ^1^, Ludger Schöls ^3,5^ and Peter Bauer ^1^

^1^ Institute of Medical Genetics and Applied Genomics, University of Tübingen, Germany

^2^ Institute of Human Genetics, University Medical Center Freiburg, Germany

^3^ Clinical Neurogenetics, Department of Neurology and Hertie-Institute for Clinical Brain Research, University of Tübingen, Tübingen, Germany

^4^ Department of Neurology, University of Mainz, Germany

^5^ German Center for Neurodegenerative Diseases (DZNE), Tübingen, Germany

*N.A. Schlipf and R. Schüle contributed equally to this work

**Supplementary Material**

Supplementary table 1: Primers for all coding exons and intron-boundaries of the *AP5Z1* gene (RefSeq: NM_014855.2).

| exon | primer | sequence 5´-> 3´ | fragment length |
| --- | --- | --- | --- |
| *AP5Z1* Exon01 | 1F | M13-ACGCAGCCACGTAAGGC | 282 bp |
|  | 1R | revM13-GAGTCCAGTCGGGTTCCTG |  |
| *AP5Z1* Exon02 | 2F | M13-TCCCTGCTCCAAGGGTTATC | 277 bp |
|  | 2R | revM13-GGGCTGGGTTCTCCTGG |  |
| *AP5Z1* Exon03 | 3F | M13-GTGCTCCTGCCACGGTC | 300 bp |
|  | 3R | revM13-CTGTTCCTTGGGAAGCAGC |  |
| *AP5Z1* Exon04 | 4F | M13-gctggtctctggcacagg | 250 bp |
|  | 4R | revM13-aggacctgggaaggcaga |  |
| *AP5Z1* Exon05 | 5F | M13-acaacccaggcatctgtagg | 199 bp |
|  | 5R | revM13-cccatggttccagaacagtc |  |
| *AP5Z1* Exon06 | 6F | M13-GGAGCTTGTGCTAAAGGCTG | 306 bp |
|  | 6R | revM13-CGACCAACCCATAGTGCG |  |
| *AP5Z1* Exon07 | 7F | M13-CAGCAGGCATGTCCCAG | 272 bp |
|  | 7R | revM13-CTCAGGTGGCCTTGCAG |  |
| *AP5Z1* Exon08-09 | 8-9F | M13-ctcattgggccactctaagg | 382 bp |
|  | 8-9R | revM13-aaactaaaggcagaagcagagc |  |
| *AP5Z1* Exon10 | 10F | M13-CCCTAACCAGTCACAGAAGC | 353 bp |
|  | 10R | revM13-CTATGCTCAGCCAGGACTCG |  |
| *AP5Z1* Exon11 | 11F | M13-CCTAGCTGGCTCCTCCCTC | 280 bp |
|  | 11R | revM13-GGGCCCAGTGTGTGGAAC |  |
| *AP5Z1* Exon12 | 12F | M13-GTGCCCTTGAGTGCAGG | 280 bp |
|  | 12R | revM13-GGCTGAGCAGTGAGCCC |  |
| *AP5Z1* Exon13 | 13F | M13-GCCTGCAGTCACCAGGTC | 265 bp |
|  | 13R | revM13-TTCTGCAGAGCTCAAGAGGG |  |
| *AP5Z1* Exon14 | 14F | M13-CCTCACCATGGCTTCACC | 229 bp |
|  | 14R | revM13-AGTCCAGGAGGCACCCG |  |
| *AP5Z1* Exon15-16 | 15-16F | M13-gcacagagcaggcgtagac | 489 bp |
|  | 15-16R | revM13-cttcgctccctccctgac |  |
| *AP5Z1* Exon17 | 17F | M13-AGCTAAAGCCACTCTGCTGG | 464 bp |
|  | 17R | revM13-GCCCTCCTGAGCTCCTATC |  |

**Second PCR with adaptor-barcode-primer**

To attach the required sequencing adaptor (A and B) for Roche 454 Titanium protocol a second PCR with custom sequencing-adaptor-barcode-primers was performed on a Thermocycler G-Storm GS1 (Alpha Metrix Biotech GmbH, Rödermark, Germany). Primer sequences are available in supplementary table2. All pooled *AP5Z1* specific fragments were diluted 1:10 and 1 µl product was used as template for the second PCR. The reaction mixture containing 2 µM of both forward and reverse primer, 10x FastStart High Fidelity Reaction Buffer with 18 mM MgCl_2_ (Roche), 1 U FastStart High Fidelity Enzyme Blend (Roche), 3% DMSO (Roche), 60 µM of each dNTP (PCR Grade Nucleotide Mix, Roche) and PCR grade water to a volume of 15 µl. The cycling conditions included an activation step at 95°C for 10 minutes followed by 10 cycles 95°C for 15 seconds, annealing at 60°C for 30 seconds and extension at 72°C during 60 seconds.

Supplementary table 2: Lib-A adaptor-barcode-primer

| adaptor-barcode-primer | sequence 5´-> 3´ |
| --- | --- |
| LibA A-Adaptor-MID1-M13 | CGTATCGCCTCCCTCGCGCCATCAGACGAGTGCGT-M13 |
| LibA B-Adaptor-MID1-revM13 | CTATGCGCCTTGCCAGCCCGCTCAGACGAGTGCGT-revM13 |
| LibA A-Adaptor-MID2-M13 | CGTATCGCCTCCCTCGCGCCATCAGACGCTCGACA-M13 |
| LibA B-Adaptor-MID2-revM13 | CTATGCGCCTTGCCAGCCCGCTCAGACGCTCGACA-revM13 |
| LibA A-Adaptor-MID3-M13 | CGTATCGCCTCCCTCGCGCCATCAGAGACGCACTC-M13 |
| LibA B-Adaptor-MID3-revM13 | CTATGCGCCTTGCCAGCCCGCTCAGAGACGCACTC-revM13 |
| LibA A-Adaptor-MID4-M13 | CGTATCGCCTCCCTCGCGCCATCAGAGCACTGTAG-M13 |
| LibA B-Adaptor-MID4-revM13 | CTATGCGCCTTGCCAGCCCGCTCAGAGCACTGTAG-revM13 |
| LibA A-Adaptor-MID5-M13 | CGTATCGCCTCCCTCGCGCCATCAGATCAGACACG-M13 |
| LibA B-Adaptor-MID5-revM13 | CTATGCGCCTTGCCAGCCCGCTCAGATCAGACACG-revM13 |
| LibA A-Adaptor-MID6-M13 | CGTATCGCCTCCCTCGCGCCATCAGATATCGCGAG-M13 |
| LibA B-Adaptor-MID6-revM13 | CTATGCGCCTTGCCAGCCCGCTCAGATATCGCGAG-revM13 |
| LibA A-Adaptor-MID7-M13 | CGTATCGCCTCCCTCGCGCCATCAGCGTGTCTCTA-M13 |
| LibA B-Adaptor-MID7-revM13 | CTATGCGCCTTGCCAGCCCGCTCAGCGTGTCTCTA-revM13 |
| LibA A-Adaptor-MID8-M13 | CGTATCGCCTCCCTCGCGCCATCAGCTCGCGTGTC-M13 |
| LibA B-Adaptor-MID8-revM13 | CTATGCGCCTTGCCAGCCCGCTCAGCTCGCGTGTC-revM13 |
| LibA A-Adaptor-MID9-M13 | CGTATCGCCTCCCTCGCGCCATCAGTAGTATCAGC-M13 |
| LibA B-Adaptor-MID9-revM13 | CTATGCGCCTTGCCAGCCCGCTCAGTAGTATCAGC-revM13 |
| LibA A-Adaptor-MID10-M13 | CGTATCGCCTCCCTCGCGCCATCAGTCTCTATGCG-M13 |
| LibA B-Adaptor-MID10-revM13 | CTATGCGCCTTGCCAGCCCGCTCAGTCTCTATGCG-revM13 |
| LibA A-Adaptor-MID11-M13 | CGTATCGCCTCCCTCGCGCCATCAGTGATACGTCT-M13 |
| LibA B-Adaptor-MID11-revM13 | CTATGCGCCTTGCCAGCCCGCTCAGTGATACGTCT-revM13 |
| LibA A-Adaptor-MID12-M13 | CGTATCGCCTCCCTCGCGCCATCAGTACTGAGCTA-M13 |
| LibA B-Adaptor-MID12-revM13 | CTATGCGCCTTGCCAGCCCGCTCAGTACTGAGCTA-revM13 |
| LibA A-Adaptor-MID13-M13 | CGTATCGCCTCCCTCGCGCCATCAGCATAGTAGTG-M13 |
| LibA B-Adaptor-MID13-revM13 | CTATGCGCCTTGCCAGCCCGCTCAGCATAGTAGTG-revM13 |
| LibA A-Adaptor-MID14-M13 | CGTATCGCCTCCCTCGCGCCATCAGCGAGAGATAC-M13 |
| LibA B-Adaptor-MID14-revM13 | CTATGCGCCTTGCCAGCCCGCTCAGCGAGAGATAC-revM13 |
| LibA A-Adaptor-MID15-M13 | CGTATCGCCTCCCTCGCGCCATCAGATACGACGTA-M13 |
| LibA B-Adaptor-MID15-revM13 | CTATGCGCCTTGCCAGCCCGCTCAGATACGACGTA-revM13 |
| LibA A-Adaptor-MID16-M13 | CGTATCGCCTCCCTCGCGCCATCAGTCACGTACTA-M13 |
| LibA B-Adaptor-MID16-revM13 | CTATGCGCCTTGCCAGCCCGCTCAGTCACGTACTA-revM13 |
| LibA A-Adaptor-MID17-M13 | CGTATCGCCTCCCTCGCGCCATCAGCGTCTAGTAC-M13 |
| LibA B-Adaptor-MID17-revM13 | CTATGCGCCTTGCCAGCCCGCTCAGCGTCTAGTAC-revM13 |
| LibA A-Adaptor-MID18-M13 | CGTATCGCCTCCCTCGCGCCATCAGTCTACGTAGC-M13 |
| LibA B-Adaptor-MID18-revM13 | CTATGCGCCTTGCCAGCCCGCTCAGTCTACGTAGC-revM13 |
| LibA A-Adaptor-MID19-M13 | CGTATCGCCTCCCTCGCGCCATCAGTGTACTACTC-M13 |
| LibA B-Adaptor-MID19-revM13 | CTATGCGCCTTGCCAGCCCGCTCAGTGTACTACTC-revM13 |
| LibA A-Adaptor-MID20-M13 | CGTATCGCCTCCCTCGCGCCATCAGACGACTACAG-M13 |
| LibA B-Adaptor-MID20-revM13 | CTATGCGCCTTGCCAGCCCGCTCAGACGACTACAG-revM13 |
| LibA A-Adaptor-MID21-M13 | CGTATCGCCTCCCTCGCGCCATCAGCGTAGACTAG-M13 |
| LibA B-Adaptor-MID21-revM13 | CTATGCGCCTTGCCAGCCCGCTCAGCGTAGACTAG-revM13 |
| LibA A-Adaptor-MID22-M13 | CGTATCGCCTCCCTCGCGCCATCAGTACGAGTATG-M13 |
| LibA B-Adaptor-MID22-revM13 | CTATGCGCCTTGCCAGCCCGCTCAGTACGAGTATG-revM13 |
| LibA A-Adaptor-MID23-M13 | CGTATCGCCTCCCTCGCGCCATCAGTACTCTCGTG-M13 |
| LibA B-Adaptor-MID23-revM13 | CTATGCGCCTTGCCAGCCCGCTCAGTACTCTCGTG-revM13 |
| LibA A-Adaptor-MID24-M13 | CGTATCGCCTCCCTCGCGCCATCAGTAGAGACGAG-M13 |
| LibA B-Adaptor-MID24-revM13 | CTATGCGCCTTGCCAGCCCGCTCAGTAGAGACGAG-revM13 |
| LibA A-Adaptor-MID25-M13 | CGTATCGCCTCCCTCGCGCCATCAGTCGTCGCTCG-M13 |
| LibA B-Adaptor-MID25-revM13 | CTATGCGCCTTGCCAGCCCGCTCAGTCGTCGCTCG-revM13 |
| LibA A-Adaptor-MID26-M13 | CGTATCGCCTCCCTCGCGCCATCAGACATACGCGT-M13 |
| LibA B-Adaptor-MID26-revM13 | CTATGCGCCTTGCCAGCCCGCTCAGACATACGCGT-revM13 |
| LibA A-Adaptor-MID27-M13 | CGTATCGCCTCCCTCGCGCCATCAGACGCGAGTAT-M13 |
| LibA B-Adaptor-MID27-revM13 | CTATGCGCCTTGCCAGCCCGCTCAGACGCGAGTAT-revM13 |
| LibA A-Adaptor-MID28-M13 | CGTATCGCCTCCCTCGCGCCATCAGACTACTATGT-M13 |
| LibA B-Adaptor-MID28-revM13 | CTATGCGCCTTGCCAGCCCGCTCAGACTACTATGT-revM13 |
| LibA A-Adaptor-MID29-M13 | CGTATCGCCTCCCTCGCGCCATCAGACTGTACAGT-M13 |
| LibA B-Adaptor-MID29-revM13 | CTATGCGCCTTGCCAGCCCGCTCAGACTGTACAGT-revM13 |
| LibA A-Adaptor-MID30-M13 | CGTATCGCCTCCCTCGCGCCATCAGAGACTATACT-M13 |
| LibA B-Adaptor-MID30-revM13 | CTATGCGCCTTGCCAGCCCGCTCAGAGACTATACT-revM13 |
| LibA A-Adaptor-MID31-M13 | CGTATCGCCTCCCTCGCGCCATCAGAGCGTCGTCT-M13 |
| LibA B-Adaptor-MID31-revM13 | CTATGCGCCTTGCCAGCCCGCTCAGAGCGTCGTCT-revM13 |
| LibA A-Adaptor-MID32-M13 | CGTATCGCCTCCCTCGCGCCATCAGAGTACGCTAT-M13 |
| LibA B-Adaptor-MID32-revM13 | CTATGCGCCTTGCCAGCCCGCTCAGAGTACGCTAT-revM13 |
| LibA A-Adaptor-MID33-M13 | CGTATCGCCTCCCTCGCGCCATCAGATAGAGTACT-M13 |
| LibA B-Adaptor-MID33-revM13 | CTATGCGCCTTGCCAGCCCGCTCAGATAGAGTACT-revM13 |
| LibA A-Adaptor-MID34-M13 | CGTATCGCCTCCCTCGCGCCATCAGCACGCTACGT-M13 |
| LibA B-Adaptor-MID34-revM13 | CTATGCGCCTTGCCAGCCCGCTCAGCACGCTACGT-revM13 |
| LibA A-Adaptor-MID35-M13 | CGTATCGCCTCCCTCGCGCCATCAGCAGTAGACGT-M13 |
| LibA B-Adaptor-MID35-revM13 | CTATGCGCCTTGCCAGCCCGCTCAGCAGTAGACGT-revM13 |
| LibA A-Adaptor-MID36-M13 | CGTATCGCCTCCCTCGCGCCATCAGCGACGTGACT-M13 |
| LibA B-Adaptor-MID36-revM13 | CTATGCGCCTTGCCAGCCCGCTCAGCGACGTGACT-revM13 |
| LibA A-Adaptor-MID37-M13 | CGTATCGCCTCCCTCGCGCCATCAGTACACACACT-M13 |
| LibA B-Adaptor-MID37-revM13 | CTATGCGCCTTGCCAGCCCGCTCAGTACACACACT-revM13 |
| LibA A-Adaptor-MID38-M13 | CGTATCGCCTCCCTCGCGCCATCAGTACACGTGAT-M13 |
| LibA B-Adaptor-MID38-revM13 | CTATGCGCCTTGCCAGCCCGCTCAGTACACGTGAT-revM13 |
| LibA A-Adaptor-MID39-M13 | CGTATCGCCTCCCTCGCGCCATCAGTACAGATCGT-M13 |
| LibA B-Adaptor-MID39-revM13 | CTATGCGCCTTGCCAGCCCGCTCAGTACAGATCGT-revM13 |
| LibA A-Adaptor-MID40-M13 | CGTATCGCCTCCCTCGCGCCATCAGTACGCTGTCT-M13 |
| LibA B-Adaptor-MID40-revM13 | CTATGCGCCTTGCCAGCCCGCTCAGTACGCTGTCT-revM13 |
| LibA A-Adaptor-MID41-M13 | CGTATCGCCTCCCTCGCGCCATCAGTAGTGTAGAT-M13 |
| LibA B-Adaptor-MID41-revM13 | CTATGCGCCTTGCCAGCCCGCTCAGTAGTGTAGAT-revM13 |
| LibA A-Adaptor-MID42-M13 | CGTATCGCCTCCCTCGCGCCATCAGTCGATCACGT-M13 |
| LibA B-Adaptor-MID42-revM13 | CTATGCGCCTTGCCAGCCCGCTCAGTCGATCACGT-revM13 |
| LibA A-Adaptor-MID43-M13 | CGTATCGCCTCCCTCGCGCCATCAGTCGCACTAGT-M13 |
| LibA B-Adaptor-MID43-revM13 | CTATGCGCCTTGCCAGCCCGCTCAGTCGCACTAGT-revM13 |
| LibA A-Adaptor-MID44-M13 | CGTATCGCCTCCCTCGCGCCATCAGTCTAGCGACT-M13 |
| LibA B-Adaptor-MID44-revM13 | CTATGCGCCTTGCCAGCCCGCTCAGTCTAGCGACT-revM13 |
| LibA A-Adaptor-MID45-M13 | CGTATCGCCTCCCTCGCGCCATCAGTCTATACTAT-M13 |
| LibA B-Adaptor-MID45-revM13 | CTATGCGCCTTGCCAGCCCGCTCAGTCTATACTAT-revM13 |
| LibA A-Adaptor-MID46-M13 | CGTATCGCCTCCCTCGCGCCATCAGTGACGTATGT-M13 |
| LibA B-Adaptor-MID46-revM13 | CTATGCGCCTTGCCAGCCCGCTCAGTGACGTATGT-revM13 |
| LibA A-Adaptor-MID47-M13 | CGTATCGCCTCCCTCGCGCCATCAGTGTGAGTAGT-M13 |
| LibA B-Adaptor-MID47-revM13 | CTATGCGCCTTGCCAGCCCGCTCAGTGTGAGTAGT-revM13 |
| LibA A-Adaptor-MID48-M13 | CGTATCGCCTCCCTCGCGCCATCAGACAGTATATA-M13 |
| LibA B-Adaptor-MID48-revM13 | CTATGCGCCTTGCCAGCCCGCTCAGACAGTATATA-revM13 |

**Data Analysis and Coverage**

Sequence Pilot Software module SeqNext Version 3.5.0 Build 504 (JSI medical systems GmbH, Kippenheim, Germany) was used for mapping and base calling of the datasets. Additionally, coverage statistics were extracted from the SeqNext modul. To evaluate the performance of our approach, the mean coverage across all analyzed regions of the *AP5Z1* gene (coding exons + min. 20 bp flanking intronic sequence) was calculated for each sample. Coverage warning levels were set to 'low coverage' for less than 10 reads and 'dropout' for complete failure. For all fragments with “low coverage” (less than 10 reads coverage), Sanger sequencing was performed.

**Detection and Prioritization of Variants**

For the detection of variants, filters were set to display sequence variances occurring in more than 10% of bidirectional reads. SeqNext software annotated high-quality differences with known polymorphism data (http://www.ncbi.nlm.nih.gov/SNP), genomic feature annotation (exon, intron and UTR-regions) and amino acid translation. Due to the recessive mode of inheritance, sequence variants obtained with NGS amplicon sequencing were filtered at the following scheme: (1) exclusion of known dbSNPs; (2) inclusion of sequence variants in coding regions and exon/intron boundaries; (3) inclusion of homozygous variants or compound heterozygous variants. Additionally, *in silico* evidence was evaluated by missense prediction programs (Polyphen and Alamut), splice site prediction (BDGP), phosphorylation site prediction (NetPhos 2.0) and evolutionary conservation scores (scorePhastCons; UCSC Genome Browser and scoreGERP; Genomic Evolutionary Rate Profiling, Sidow Lab). Also, we established the frequencies of putative mutations: (1) within in-house Caucasian control chromosomes, and/or (2) in the latest release of the NHLBI Exome Sequencing Project (Exome Variant Server; http://evs.gs.washington.edu/EVS/) and in the 1000 genomes project (www.1000genomes.org). Variant validation was performed using Sanger sequencing.

**Copy Number Variation Analysis**

To detect possible deletions and duplications within the *AP5Z1* gene we used the CNV tool of SeqNext. Moreover, we designed a multiplex ligation-dependent probe amplification (MLPA) assay, which targets the coding sequence of 7 exons, with one probe for each exon. Four probes localizing to different chromosomes were included as controls. Conformable synthetic oligonucleotides (Metabion GmbH, Martinsried, Germany) and reagents from the EK5 kit provided by MRC-Holland (Amsterdam, The Netherlands) were used according to the instructions of the manufacturers.

Supplementary Figure S1:


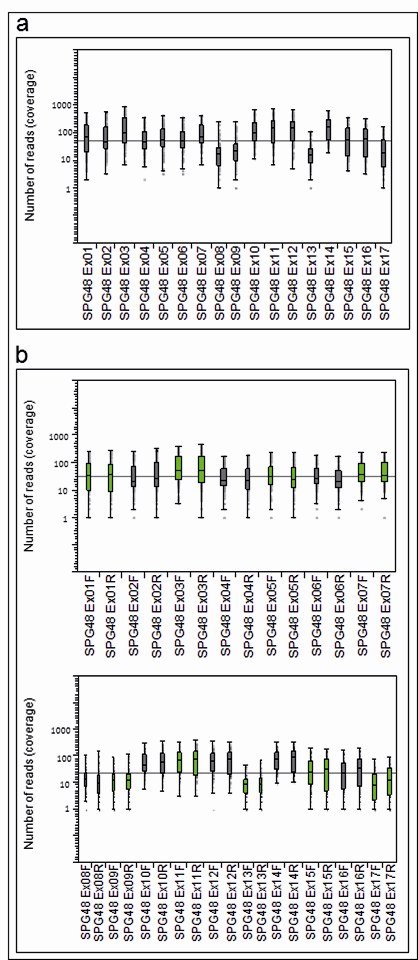


**Figure S1: Coverage distribution across amplicons**

(a) For each of the amplicons (x-axis), the distribution of generated reads is represented (y-axis). Box and –whiskers plots summarize the corresponding overall coverage and (b) according to forward (A reads) and and reverse (B reads). (*AP5Z1* RefSeq: NM_014855.2).

Supplementary Figure S2:


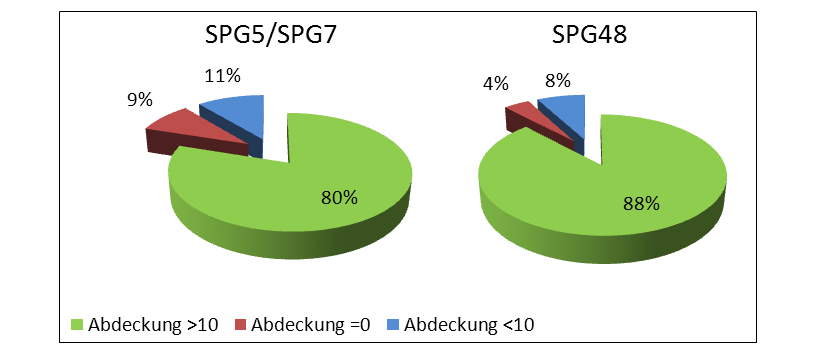


**Figure S2: Performance of the study**

In total 88% of the amplicons (green, 1899 of 2159) were covered successfully (>10-fold). Dropouts of single amplicons with no coverage were obtained in 91 of 2159 amplicons (red, 4%) and 8 % of the amplicons (blue, 169 of 2159) were insufficiently covered less than 10-fold and were completed by conventional Sanger sequencing. (*AP5Z1* RefSeq: NM_014855.2).

Supplementary table 3: Sequence variants found in *AP5Z1*

| **mutation at DNA level; protein level** | **location** | **heterozygous patients/**  **homozygous patients** | **frequency sample chromosomes** | **frequency control chromosomes** | **Exome Variant Server (allele frequency)** | **Predicted effect**** |
| --- | --- | --- | --- | --- | --- | --- |
| c.281C>G; p.S94C | 3 | 5/0 | 5/254 (1.9%) | n.a. | 220/12552 = 0,017 (1.7%) | known polymorphism, rs11549839 |
| c.333G>C; p.Q111H | 3 | 5/0 | 5/254 (1.9%) | n.a. | 218/12550 = 0,017 (1.7%) | known polymorphism, rs11549840 |
| c.237C>T; p.P79P | 3 | 1/0 | 1/254 (0.4%) | n.a. | not reported | no indication for effect on splicing |
| c.417G>A; p.A139A | 4 | 1/0 | 1/254 (0.4%) | n.a. | 1/12555 = 0,00008 (0.008%) | no indication for effect on splicing |
| c.490A>G; p.S164G | 4 | 1/0 | 1/254 (0.4%) | 1/266 (0.4%) | not reported | benign, no indication for effect on splicing |
| c.-7A>G | 5 | 1/0 | 1/254 (0.4%) | n.a. | 695/10651 = 0,065 (6.5%) | known polymorphism, rs73305371 |
| c.588C>T; p.S196S | 5 | 1/0 | 1/254 (0.4%) | n.a. | 1/11663 = 0,00008 (0.008%) | known polymorphism, rs146665638 |
| c.729C>T; p.F243F | 6 | 1/0 | 1/254 (0.4%) | n.a. | not reported | no indication for effect on splicing |
| c.759C>T; p.S253S | 6 | 19/0 | 19/254 (7.1%) | n.a. | 2686/9620 = 0,28 (27.9%) | known polymorphism, rs17135121 |
| c.780C>A; p.T260T | 6 | 1/0 | 1/254 (0.4%) | n.a. | 4/11926 = 0,0003 (0.03%) | no indication for effect on splicing |
| c.-5C>T | 7 | 1/0 | 1/254 (0.4%) | n.a. | 49/12353 = 0,004 (0.4%) | known polymorphism, rs73671921 |
| c.1124T>A; p.L375Q | 9 | 14/1 | 16/254 (6.0%) | n.a. | 600/11942 = 0,05 (5.02%) | known polymorphism, rs11772411 |
| c.1131C>T; p.H377H | 9 | 14/1 | 16/254 (6.0%) | n.a. | 607/11961 = 0,05 (5.07%) | known polymorphism, rs11768079 |
| c.-13C>T | 9 | 1/0 | 1/254 (0.4%) | n.a. | 913/12025 = 0,076 (7.6%) | known polymorphism, rs73305376 |
| c.1161C>T; p.A387A | 10 | 2/0 | 2/254 (0.8%) | n.a. | 1/12285 = 0,00008 (0.008%) | no indication for effect on splicing |
| c.1197G>A; p.E399E* | 10 | 4/0 | 4/254 (1.5%) | n.a. | 217/12143 = 0,018 (1.8%) | polymorphism* |
| c.1290G>A; p.L430L | 10 | 1/0 | 1/254 (0.4%) | n.a. | not reported | no indication for effect on splicing |
| c.1377C>T; p.D459D | 11 | 16/0 | 16/254 (6.0%) | n.a. | 721/11723 = 0,06 (6.1%) | known polymorphism, rs17135128 |
| c.1529G>A; p.R510Q | 12 | 5/0 | 5/254 (1.9%) | n.a. | 209/11942 = 0,02 (1.75%) | known polymorphism, rs77890266 |
| c.1554C>T; p.F518F | 12 | 1/0 | 1/254 (0.4%) | n.a. | 237/11717 = 0,02 (2.0%) | known polymorphism, rs77560694 |
| c.1573A>C; p.K525Q* | 12 | 2/0 | 2/254 (0.8%) | n.a. | not reported | variant of unknown function |
| c.+11T>C | 13 | 17/1 | 19/254 (7.4%) | n.a. | 2054/10398 = 0,197 (19.7%) | known polymorphism, rs3750012 |
| c.2098G>A; p.V700M | 16 | 3/0 | 3/254 (1.1%) | n.a. | 185/12245 = 0,015 (1.5%) | known polymorphism, rs11766611 |
| c.-9C>T | 16 | 1/0 | 1/254 (0.4%) | n.a. | 520/11696 = 0,044 (4.44%) | known polymorphism, rs73305392 |
| c.2274G>A; p.L758L | 17 | 1/0 | 1/254 (0.4%) | n.a. | not reported | no indication for effect on splicing |
| c.2379G>T; p.T793T | 17 | 5/0 | 5/254(1.9%) | n.a. | 216/12228 =0,017 (1.76%) | known polymorphism, rs77393809 |
| c.2400G>A; p.R800R | 17 | 1/0 | 1/254 (0.4%) | n.a. | 3/12395 = 0,0002 (0.2%) | no indication for effect on splicing |
| 3'UTR | 17 | 9/0 | 9/254 (3.5%) | n.a. | 833/11437 = 0,072 (7.2%) | known polymorphism, rs12154545 |

* Slabicki, M., et al., A genome-scale DNA repair RNAi screen identifies SPG48 as a novel gene associated with hereditary spastic paraplegia. PLoS Biol, 2010. **8**(6): p. e1000408.

** phosphorylation site prediction: NetPhos 2.0, prediction of effect on protein function: PolyPhen, splice site prediction: BDGP

*AP5Z1* RefSeq: NM_014855.2
